# Left atrial appendage occlusion should be offered only to select atrial fibrillation patients

**DOI:** 10.1016/j.hroo.2022.07.001

**Published:** 2022-08-22

**Authors:** Muhammad Bilal Munir, Jonathan C. Hsu

**Affiliations:** ∗ Section of Electrophysiology, Division of Cardiology, University of California Davis, Sacramento, California; † Section of Electrophysiology, Division of Cardiology, University of California San Diego, La Jolla, California

**Keywords:** Left atrial appendage occlusion, Subgroups, Oral anticoagulants, Direct-acting oral anticoagulants, Women, Kidney disease, Heart failure

**Figure:**
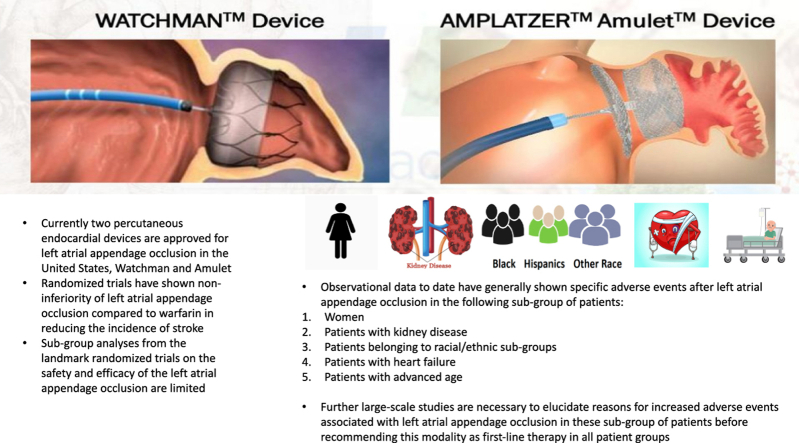

**Key Findings**

▪ Percutaneous left atrial appendage (LAA) occlusion has emerged as an alternative strategy to oral anticoagulants in selected patients with atrial fibrillation.
▪ The landmark trials comparing LAA occlusion to an oral anticoagulation strategy enrolled patients with no apparent contraindications to the use of warfarin.
▪ LAA occlusion has limited head-to-head comparison against the direct-acting oral anticoagulants.
▪ Observational data to date have generally shown specific adverse events after LAA occlusion in specific subgroups of patients (women, patients with kidney disease and heart failure, patients belonging to racial/ethnic subgroups and with advanced age), but further large-scale studies are necessary to elucidate reasons for increased adverse events associated with LAA occlusion in these subgroups of patients before recommending this modality as first-line therapy in all patient groups.

## Introduction

Atrial fibrillation (AF) is the most prevalent arrhythmia encountered in clinical practice and is associated with significant morbidity and mortality.^1^^,^^2^ One of the most severe complications of AF is stroke, and AF associated strokes tend to be more disabling when compared to non-AF-related strokes.^3^^,^^4^ Oral anticoagulants (OACs) are currently the standard of care for mitigation of ischemic stroke risk in AF patients.^5^, ^6^, ^7^, ^8^ However, nearly half of eligible AF patients do not receive OACs, owing to a multitude of factors.^9^, ^10^, ^11^, ^12^, ^13^ Approximately 90% of intracardiac thrombi originate in the left atrial appendage in AF patients.^14^ Recently, percutaneous left atrial appendage occlusion (LAAO) has been shown to be effective in minimizing the stroke risk in AF patients.^15^, ^16^, ^17^, ^18^ The 2 currently approved endocardial devices used for LAAO in the United States are the Watchman (Boston Scientific, Marlborough, MA) and Amplatzer Amulet (Abbott, Chicago, IL).^17^^,^^18^ In the PROTECT AF trial,^15^ LAAO using an earlier-generation Watchman device (Boston Scientific, Marlborough, MA) was found to be noninferior to warfarin in reducing the incidence of stroke. The more recently conducted PINNACLE FLX trial^17^ further corroborated the safety and efficacy of the newer-generation Watchman FLX device (Boston Scientific, Marlborough, MA) in reducing the risk of stroke in AF patients. The Amulet IDE trial^18^ also showed noninferiority of the Amplatzer Amulet device (Abbott, Chicago, IL) in reducing the stroke risk compared to the first-generation Watchman device (Boston Scientific, Marlborough, MA).

In this viewpoint, we will respectfully present our perspective on why percutaneous LAAO should be offered only to selected AF patients for reduction of stroke risk by highlighting the significant methodological issues with the conduction of landmark PROTECT AF and PREVAIL trials, absence of comparable efficacy and safety data for percutaneous LAAO with respect to direct-acting oral anticoagulants (DOACs), and largely nonsupportive observational evidence for percutaneous LAAO implantation in certain subgroups of patients.

### Methodological issues with the PROTECT AF and PREVAIL trials

The PROTECT AF and PREVAIL trials were the first randomized comparisons between percutaneous LAAO using an earlier-generation Watchman device (Boston Scientific, Marlborough, MA) and warfarin.^15^^,^^16^ Several important methodological issues pertinent to the conduct of these trials need to be highlighted (Table 1). Both trials used a Bayesian method to determine the outcomes instead of a traditional frequentist method. Bayesian methodology incorporates prior probabilities in the statistical analysis and updates this probability as events accumulate.^19^, ^20^, ^21^, ^22^ In this way investigators can reach the conclusion faster and with a small sample size. Therefore, Bayesian design is more flexible to determine the treatment effect but is inherently prone to extrapolate false-positive conclusions about the outcomes of interest.^22^ Both trials used a wider noninferiority rate ratio margin for the primary efficacy endpoint (2 for the PROTECT AF trial and 1.75 for the PREVAIL trial) when compared to the earlier trials on DOACs.^5^, ^6^, ^7^, ^8^ Additionally, the PROTECT AF trial did not have a prespecified noninferiority margin for the primary safety endpoint. Both the PROTECT AF and PREVAIL trials incorporated “cardiovascular/unexplained death” as a component of the composite primary efficacy endpoint (the other 2 components were stroke and systemic embolism). Earlier trials studying DOACs only adjudicated stroke and systemic embolism as part of the primary efficacy endpoint and did not include cardiovascular/unexplained death. While it may be reasonable to include outcomes such as cardiovascular/unexplained death as an endpoint in the PROTECT AF and PREVAIL trials owing to procedural safety concerns, it also made noninferiority easier to reach for the LAAO arm, as this outcome was not frequent in both trial arms.^23^ The LAAO arm of the PROTECT AF trial had increased embolic stroke events (2.2 per 100 patient-years) when compared to the warfarin arm (1.6 per 100 patient-years), while the rate of hemorrhagic stroke was lower in the LAAO arm (0.1 per 100 patient-years) in comparison to the warfarin arm (1.6 per 100 patient-years). It is important that both referring and implanting physicians are aware of important limitations of the pivotal LAAO trials so they can best inform decisions on optimal patient selection for percutaneous LAAO.

**Table 1:** Summary of important trials of percutaneous left atrial appendage occlusion and associated limitations

Trial	Study arms	Sample size	Outcomes of interest	Results	Important limitations
PROTECT AF^15^	LAAO using first-generation Watchman vs warfarin, 2:1 randomization, noninferiority study design	707	(1) Primary efficacy endpoint = composite of stroke, SE, and CV/unexplained deaths (2) Primary safety endpoint = composite of significant bleeding or procedure-related complications (serious pericardial effusion, device embolization, and procedure-related stroke)	(1) LAAO noninferior for the efficacy endpoint (95% credible interval 0.35–1.25, criteria for noninferiority <2) (2) High rate of significant pericardial effusion (4.8%), procedural stroke (1.1%), and embolization (0.6%) in the LAAO arm	(1) Bayesian framework, cannot rule out false-positive conclusions for the outcomes of interest (2) Wider noninferiority margin of 2 (3) High rate of procedural complications, especially serious pericardial effusion (4) No prespecified noninferiority margin for the primary safety endpoint
PREVAIL^16^	LAAO using first-generation Watchman vs warfarin, 2:1 randomization, noninferiority study design	407	(1) First primary efficacy endpoint = composite of all stroke, SE, and CV/unexplained deaths (2) Second primary efficacy endpoint = composite of ischemic stroke and SE 7 days after implantation (3) Primary safety endpoint = composite of all-cause death, ischemic stroke, SE, and procedure-related complications within 7 days of implantation	(1) LAAO was inferior for the first primary efficacy endpoint (95% credible interval 0.57–1.89, criteria for noninferiority <1.75) (2) LAAO was noninferior for the second primary efficacy endpoint (rate difference -0.0190 to 0.0273, criteria for noninferiority <0.0275) (3) Safety events 2.2% in the LAAO arm	(1) Did not reach noninferiority for the first primary efficacy endpoint (2) Bayesian framework, cannot rule out false-positive conclusions for the outcomes of interest (3) Wider noninferiority margin of 1.75
PINNACLE FLX^17^	Single arm (LAAO using Watchman FLX)	400	(1) Primary efficacy endpoint = effective closure (device leak of ≤5 mm at 1 year) (2) Primary safety endpoint = death, ischemic stroke, SE, or device-related major events requiring surgery or endovascular interventions within 7 days of implant	(1) Incidence of primary efficacy endpoint was 100%, which exceeds performance goal of 97% (2) Incidence of primary safety endpoint was 0.5% with 95% upper CI of 1.6, meeting the performance goal of <4.21	(1) Single-arm study, no control group (2) The efficacy endpoint was reported as an effective seal at 1 year (≤5 mm device) and no rates of stroke and SE were reported
AMULET IDE^18^	Amulet vs first-generation Watchman, 1:1 randomization, noninferiority study design	1878	(1) Primary efficacy endpoint = composite of ischemic stroke or SE (2) Primary safety endpoint = composite of procedure-related complications, all-cause death, and major bleeding	(1) Amulet was noninferior to the Watchman device for the primary efficacy endpoint (2.8% vs 2.8%, *P* < .001 for noninferiority) (2) Amulet was noninferior to the Watchman device for the primary safety endpoint (14.5% vs 14.7%, *P* < .001 for noninferiority)	(1) Comparison was made with the first-generation Watchman device and not with newer Watchman FLX device (2) High rates of pericardial effusion (4.5%) and device embolization (2.5%) with the Amulet device
PRAGUE-17^32^	LAAO vs DOACs, 1:1 randomization, noninferiority study design	402	Primary endpoint = composite of cardioembolic events (stroke, transient ischemic attack, and SE), cardiovascular death, clinically relevant bleeding, and procedure/device-related complication	LAAO was found to be noninferior to the DOACs for the primary endpoint (hazard ratio 0.81, 95% CI 0.56–1.18, *P* = .27, noninferiority criteria were *P* < .006)	(1) Both efficacy and safety outcomes were combined in the primary endpoint (2) Clinically significant bleeding was not low in the LAAO arm compared to the DOAC arm (3) 5% of patients in the LAAO had a serious complication from the procedure

CV = cardiovascular; DOAC = direct-acting oral anticoagulant; LAAO = left atrial appendage occlusion; SE = systemic embolism.

### Perspective on LAAO and OACs

#### AF patients who can vs cannot tolerate OACs

The PROTECT AF and PREVAIL trials compared efficacy and safety of percutaneous LAAO versus warfarin, and enrolled patients with no apparent contraindications to long-term warfarin use.^15^^,^^16^ LAAO is an attractive alternative for AF patients who cannot tolerate OACs long term and thus remain at heightened stroke risk. There are currently no randomized data evaluating the efficacy and safety of LAAO in patients with a strict contraindication to any OAC therapy. In a multicenter registry of 150 AF patients with a mean CHA_2_DS_2_-VASc score of 4.4 ± 1.7 and ineligibility for warfarin therapy, Reddy and colleagues^24^ showed that LAAO using a Watchman device conferred 64% reduction in the risk of ischemic stroke when compared to a similar risk-matched AF cohort on aspirin and clopidogrel. In a large European multicenter EWOLUTION registry analyzing more than 1000 AF patients at increased risk of stroke (with OACs contraindicated in 72%), percutaneous LAAO was found to reduce the risk of ischemic stroke by 83% at 2 years of follow-up.^25^ The 2 randomized trials ASAP TOO^26^ and STROKE CLOSE^27^ will provide further insights on the safety and efficacy of percutaneous LAAO in AF patients with contraindication to OACs. The ASAP TOO trial is not actively recruiting at the present time and the data on currently enrolled patients have not been publicly reported as of yet. LAAO is a technically challenging procedure to perform, with an associated learning curve, and although major complications associated with implantation such as pericardial effusion requiring drainage, device-related thrombus, and device embolization continue to decline in contemporary practice, such complications are associated with extensive patient morbidity and mortality.^28^^,^^29^ Additionally, the strategy of using percutaneous LAAO as an adjunct to long-term OAC therapy is not studied as of yet, but there is evidence of benefit with surgical LAAO and concomitant OAC treatment, as shown by the LAAOS III trial.^30^ Hence based on the current state of scientific evidence and especially the absence of any randomized data, the authors’ viewpoint is that percutaneous LAAO should generally be reserved for AF patients who cannot tolerate long-term anticoagulants.

#### LAAO and DOACs

The last decade has witnessed widespread assimilation of DOACs into clinical practice and they are considered first-line treatment for stroke risk reduction across a broad spectrum of AF patients.^31^ Studies have shown DOACs to be noninferior for stroke prevention and superior for bleeding risk compared to warfarin.^5–8)^ DOACs have a quick onset of action, do not require frequent laboratory monitoring, and have minimal drug- and food-related interactions. There are limited randomized data on head-to-head comparison of DOACs with percutaneous LAAO.^32^ Recently, the 4-year follow-up data from the PRAGUE-17 trial^32^ (the only randomized trial in this realm to date) was published on more than 400 AF patients who were randomized 1:1 to either a DOAC or a percutaneous LAAO. The primary endpoint of the trial was a composite of cardioembolic events (stroke, transient ischemic attack, and systemic embolism), cardiovascular death, clinically relevant bleeding, and procedure/device-related complication (for the LAAO arm). At the end of follow-up period, LAAO was found to be noninferior to the DOACs for the primary endpoint (hazard ratio 0.81, 95% confidence interval [CI] 0.56–1.18, *P* = .27; noninferiority criteria were *P* < .006). Of note, in the PRAGUE-17 trial primary endpoint was composed of both efficacy and safety outcomes, in contrast to earlier PROTECT AF and PREVAIL trials,^15^^,^^16^ and clinically relevant bleeding was not significantly better in the LAAO arm when assessed separately. In the opinion of the authors, the PRAGUE-17 trial did not demonstrate any safety advantage of percutaneous LAAO with respect to DOACs, as clinically significant bleeding was not better in the LAAO arm and approximately 5% of patients had a serious complication after LAAO device implantation. Currently, 3 large randomized trials, CHAMPION-AF,^33^ CATALYST,^34^ and Occlusion-AF,^35^ are being conducted that will evaluate the efficacy and safety of percutaneous LAAO with DOACs and will give further insight into the current topic. These trials have distinct efficacy and safety endpoints, unlike the PRAGUE-17, and both CHAMPION-AF and CATALYST trials will adjudicate safety outcomes with a superiority design framework. Furthermore, patients are enrolled in these trials based on stroke risk (as quantified by CHA_2_DS_2_-VASc score) and not by long-term DOAC ineligibility and therefore the results of these trials will inform the applicability of percutaneous LAAO to a broader AF population. Pending further evidence from these randomized controlled trials, the authors’ opinion is that DOACs should be the preferred modality of reducing stroke risk in AF patients if they are able to tolerate them without major adverse effects.

### Perspective on special patient subgroups

The randomized clinical trials evaluating the efficacy and safety of percutaneous LAAO did not stratify outcomes based on various patient subgroups. It is important to explore LAAO-related outcomes in various patient subgroups so as to identify those patients who may have differential risk from the LAAO procedure. It is necessary to point out that the authors’ opinion on these patient subgroups have largely stemmed from the observational studies, as they are not aware of any randomized data exploring LAAO-related outcomes in these patients.

#### Women

Although the age-adjusted incidence and prevalence of AF are lower in women compared to men, women generally have a higher risk of stroke and death from AF.^36^^,^^37^ The landmark PROTECT AF^15^ and PREVAIL^16^ trials evaluating the efficacy and safety of percutaneous LAAO only enrolled 30% of women patients with AF and no gender-specific subgroup analyses on outcomes were conducted in those trials. In a study of more than 49,000 patients undergoing percutaneous LAAO from the National Cardiovascular Data LAAO Registry, the authors showed that women were more likely than men to experience any adverse event related to device implantation (odds ratio [OR] 1.63, 95% CI 1.49–1.77).^38^ The prevalence of major adverse events (mostly pericardial effusion requiring drainage and major bleeding) was also higher in female patients undergoing LAAO compared to the male patients (OR 2.06, 95% CI 1.82–2.34). Additionally, we also showed that women had a longer length of hospital stay (OR 1.46, 95% CI 1.38–1.54) and mortality (OR 2.01, 95% CI 1.31–3.09) after percutaneous LAAO. Similarly, in another study of 9281 LAAO implantations from the Nationwide Readmissions Database, Osman and colleagues^39^ showed that women had a higher adjusted rate of major adverse events when compared to men (2.8% vs 1.9%, *P* < .01). They also reported a higher 30-day readmission rate after LAAO implantation in women (10% vs 8.6%, *P* = .03). Earlier studies have shown lower utilization rate of OACs among women with AF across all spectrum of stroke risk.^40^ Percutaneous LAAO should therefore be an attractive alternative in such patients. However, in lieu of the limited and generally nonsupportive scientific evidence available to date in women with respect to LAAO, detailed risk/benefit discussion is recommended to counsel on potential increased risk of adverse events from LAAO in women. There is also a dire need to explore more on the mechanisms of poor LAAO-related outcomes in female patients with a goal of making the device implantation procedure safer in women.

#### Patients with chronic and end-stage kidney disease

Chronic kidney disease (CKD) and end-stage renal disease (ESRD) are frequent comorbidities encountered in patients with AF.^41^, ^42^, ^43^ The management of anticoagulation in patients with CKD and ESRD is challenging, as these patients have simultaneous increased risk of bleeding as well as stroke.^44^, ^45^, ^46^, ^47^ Warfarin is the most studied oral anticoagulant in these groups of patients; however, it can be associated with calcific arteriolopathy (calciphylaxis), a potentially life-threatening condition.^48^ Most of the DOACs undergo some degree of renal clearance, thus making their pharmacokinetics unpredictable in patients with CKD and ESRD.^49^^,^^50^ Unfortunately, the landmark trials evaluating percutaneous LAAO have limited participation of patients with advanced kidney disease, and no subgroup analyses have been performed on such patients.^15^^,^^16^ Most of the observational data pointed to worse outcomes after percutaneous LAAO in patients with kidney disease. For example, in a study of 146 AF patients (81 with CKD; 62 with no CKD; and 3 were excluded), Brockmeyer and colleagues^51^ showed higher mortality at the conclusion of their follow-up period after LAAO device implantation in the CKD group (10.5/100 person-years vs 4.2/100 person-years). In another study of 300 patients (151 with CKD and 149 with no CKD) undergoing percutaneous LAAO implantation, Xue and colleagues^52^ also demonstrated a trend towards increased mortality in patients with CKD (15.2% vs 8.1%). In a study of more than 36,000 LAAO device implantations (3545 with CKD and 1155 with ESRD) from the National Inpatient Sample,^53^ the authors demonstrated that CKD was independently associated with prolonged length of stay (OR 1.35, 95% CI 1.23–1.49) and acute kidney injury (OR 4.13, 95% CI 3.54–4.83) while ESRD was independently associated with inpatient mortality (OR 7.16, 95% CI 3.29–15.54). The optimal stroke prevention strategy in patients with kidney disease is still controversial, and further large-scale studies are needed before LAAO can be safely recommended in such patients.

#### Patients belonging to racial/ethnic subgroups

Earlier data have shown important differences in AF-associated outcomes in patients belonging to racial/ethnic subgroups. For example, the risk of ischemic stroke is higher in Black and Hispanic AF patients on OACs when compared to White patients.^54^ The observational studies evaluating outcomes after percutaneous LAAO in patients of various racial/ethnic subgroups have shown worse outcomes in these patients. In a large national cohort of 34,960 percutaneous LAAO device implantations from years 2015–2018 in the United States,^55^ the authors demonstrated that Black patients, Hispanic patients, and patients of Other race had a higher prevalence of major complication from the procedure compared to White patients (OR 1.22, 95% CI 0.99–1.51, OR 1.3, 95% CI 1.07–1.56, and OR 1.93, 95% CI 1.57–2.36, respectively). Similarly, in another study of 16,830 percutaneous LAAO device implantations, Vincent and colleagues^56^ showed that Black patients had a higher prevalence of postprocedural stroke (0.7% vs 0.2%, *P* < .01) and bleeding requiring transfusion (4.5% vs 1.4%, *P* < .01) compared to White patients. Increased risk of adverse events with percutaneous LAAO should be noted in patients of minority races/ethnicities and further supportive data are necessary.

#### Patients with congestive heart failure

AF and congestive heart failure (CHF) frequently coexist due to similar risk factors that are involved in the pathogenesis of both clinical entities.^57^ The prevalence of CHF was 27% and 23% in the landmark PROTECT AF^15^ and PREVAIL^16^ trials, respectively. Both AF and CHF are associated with morbidity and mortality in patients when present alone; however, their combined presence amplifies mortality. The authors recently published their data of approximately 62,980 percutaneous LAAO device implantations in which they assessed postprocedural outcomes based on HF status.^58^ They demonstrated that both heart failure with preserved ejection fraction and heart failure with reduced ejection fraction were not associated with major complications and mortality but were associated with prolonged length of stay (OR 1.41, 95% CI 1.31–1.53 and OR 1.66, 95% CI 1.53–1.80) and increased hospitalization costs (OR 1.26, 95% CI 1.19–1.34 and OR 1.21, 95% CI 1.13–1.29) after percutaneous LAAO. Patients with CHF should be counseled on these potential adverse outcomes associated with LAAO and an informed decision should be made before proceeding with such implantations.

#### Patients with advanced age

In elderly patients, strokes related to AF are generally more disabling when compared to AF-associated strokes in relatively younger patients.^3^ Additionally, elderly patients are more susceptible to major bleeding when OACs are used for the reduction of stroke risk.^59^^,^^60^ Unfortunately, the trials evaluating the efficacy and safety of percutaneous LAAO included fewer elderly AF patients, and there is a paucity of observational data on outcomes after LAAO in such patients as well.^15^^,^^16^ In a study of 6779 patients undergoing percutaneous LAAO, Sanjoy and colleagues^61^ showed that older patients (≥80 years old) had a higher rate of major adverse events compared to a younger LAAO cohort (6% vs 4.6%, *P* < .01). Our own work^62^ assessed important outcomes of inpatient mortality and major complications after LAAO in patients aged ≥80 years and compared them to a cohort of patients aged <80 years. Outcomes depicted higher adjusted mortality in older patients undergoing LAAO compared to a relatively younger cohort (OR 4.44, 95% CI 2.39–8.24), but no significant adjusted risk of major complications, prolonged length of stay, and increased hospitalization costs. The authors certainly acknowledge the clinical utility of percutaneous LAAO in mitigating stroke risk in older AF patients, but would recommend further large-scale studies to assess safety of such devices in older patients.

### Cost-effectiveness of percutaneous LAAO

Few studies have evaluated cost-effectiveness of percutaneous LAAO with respect to OACs. In a study using 4-year data from the PROTECT AF trial and meta-analyses of warfarin and DOACs, Reddy and colleagues^63^ demonstrated that relative to warfarin, percutaneous LAAO was cost-effective at 7 years ($42,994/quality-adjusted life-years [QALY]), and DOACs were cost-effective at 16 years ($48,446/QALY). In another study using maximum 5-year follow-up data from the PROTECT AF and PREVAIL trials, Reddy and colleagues^64^ showed that percutaneous LAAO was cost-effective when compared to warfarin by year 7 ($48,674/QALY) and dominant (more effective and less costly) by year 10. They also showed that percutaneous LAAO became both cost-effective and dominant relative to DOACs by year 5 and remained so over the lifetime analysis.^64^ LAAO was more costly than OACs in the early years after implantation, likely secondary to procedural complications of pericardial effusion requiring intervention, stroke, device embolization, and device-related thrombus. Improvements in LAAO device safety may allow more cost-effectiveness sooner after implantation and may also result in broader acceptance across various patient groups. Furthermore, the practice of combining percutaneous LAAO and AF ablation into a single procedure has shown promising safety and efficacy and also has the potential to make both procedures cost-effective owing to minimization of inpatient resource utilization.^65^

## Conclusion

Percutaneous LAAO is an attractive alternative to OACs for reduction of stroke risk in AF patients. Much progress has been made over the last decade in making percutaneous LAAO safer and more effective for a significant proportion of patients with AF. The authors have highlighted why LAAO should not be considered the primary therapy for all AF patients, owing to robust data supporting OACs in reducing stroke risk in most AF patients, and lacking data regarding extending LAAO to all AF populations. The authors concur with the current ACC/AHA/HRS guidelines for AF management that have designated a class IIB recommendation (usefulness/efficacy less well established by evidence) for percutaneous LAAO in mitigating stroke risk in AF patients and believe that the procedure should be reserved for patients with heightened stroke risk and contraindications to long-term OACs.^66^
